# Advances in the role and mechanism of fibroblasts in fracture healing

**DOI:** 10.3389/fendo.2024.1350958

**Published:** 2024-02-26

**Authors:** Hui Wang, Li-li Qi, Clement Shema, Kui-ying Jiang, Ping Ren, He Wang, Lei Wang

**Affiliations:** ^1^ Department of Orthopedics, The First Hospital of Hebei Medical University, Shijiazhuang, Hebei, China; ^2^ Experimental Center for Teaching of Hebei Medical University, Shijiazhuang, Hebei, China; ^3^ Department of Orthopedic Research Center, The Third Hospital of Hebei Medical University, Shijiazhuang, Hebei, China; ^4^ International Education College of Hebei Medical University, Shijiazhuang, Hebei, China; ^5^ National Demonstration Center for Experimental Basic Medical Education, Capital Medical University, Beijing, China; ^6^ Department of Pathogenic Biology, Hebei Medical University, Shijiazhuang, Hebei, China; ^7^ Department of Human Anatomy, Institute of Medicine and Health, Hebei Medical University, Shijiazhuang, Hebei, China; ^8^ The Key Laboratory of Neural and Vascular Biology, Ministry of Education, Hebei Medical University, Shijiazhuang, Hebei, China; ^9^ Neuroscience Research Center, Hebei Medical University, Shijiazhuang, Hebei, China; ^10^ Hebei Key Laboratory of Neurodegenerative Disease Mechanism, Shijiazhuang, Hebei, China

**Keywords:** fibroblasts, fracture healing, bone microenvironment, cytokines, osteoblasts

## Abstract

With the development of social population ageing, bone fracture has become a global public health problem due to its high morbidity, disability and mortality. Fracture healing is a complex phenomenon involving the coordinated participation of immigration, differentiation and proliferation of inflammatory cells, angioblasts, fibroblasts, chondroblasts and osteoblasts which synthesize and release bioactive substances of extracellular matrix components, Mortality caused by age-related bone fractures or osteoporosis is steadily increasing worldwide as the population ages. Fibroblasts play an important role in the process of fracture healing. However, it is not clear how the growth factors and extracellular matrix stiffness of the bone-regeneration microenvironment affects the function of osteoblasts and fibroblasts in healing process. Therefore, this article focuses on the role of fibroblasts in the process of fracture healing and mechanisms of research progress.

## 1 Introduction

Fracture healing regenerates bone through a complex series of proliferative and morphogenic events within the injured skeletal tissues. Fractures are the most common large organ trauma in human (1). Human bone fracture has become a common public health concern globally, with 178 million new cases of fractures and 445 million cumulative cases of fractures in 2019 (2). Fractures have become a global public health problem as the number of people suffering from the incidence continues to grow from different reasons. Healing regenerates bone through a complex series of proliferative and morphogenic events within the injured skeletal, and the vast majority of fractures healed well, however the period of healing varies, depending on various factors like age, nutrients,operation process,treatment, the type of fracture and severity of the injury, and other pre-existing conditions (3).Many complex clinical conditions such as large traumatic bone defects, osteoporosis, tumor resection, or skeletal abnormalities can impair normal bone healing. Most elderly people develop osteoporosis with age. Osteoporosis results in bone loss and an increased risk of fractures that can lead to death (4, 5). Fracture healing is a complicated and slow process of repair It involves systemic or local circumstances, together with some types of cells and growth factors that antagonist works together with adjacent tissues and immunity to promote bone healing. Fracture healing can be divided into three partially overlapping phases: the inflammatory phase, the repair phase, and the remodeling phase (6). Of these, the inflammatory phase is the most critical in the overall fracture healing process, and this phase is regulated by multiple systems, including the skeletal system and the immune system (7). Fibroblasts in tissues are key cell types that regulate the activation or suppression of immune responses and play an important role in fracture healing.

Fibroblasts primarily considered as cells that support organ structures and have recently gained a privilege attention to the researcher for their roles in regulating immune responses in health and disease. Fibroblasts are not a homogenous population and show variation in gene expression and behaviors depending on the site from which the cells are isolated. Microarray analysis of genes associated with inflammation highlighted that not only do unstimulated fibroblasts have differing gene expression patterns but also that the response of fibroblasts to various stimuli varies with the site from which the cells were taken, resulting in distinct phenotypes and functions in different organs (8–10).With roles in growth and development, injury response, and tissue homeostasis, fibroblast functions within the body are dynamic and multifarious (11, 12). Fibroblasts are present in significant amounts in bone and numerous previous studies have suggested that fibroblasts have impacted in normal fracture healing (13). It is well established that fibroblast growth factors have mutagenic and angiogenic activities on mesoderm and neuroectoderm derived cells. Of particular interest as a member of the fibroblast growth factor family, basic fibroblast growth factor stimulates mitogenesis, chemotaxis, differentiation, and angiogenesis (13). Besides, recently studies have reported that fibroblast may be involve in fracture healing by regulating the interaction of immune cell and bone remodeling process (14). Although the role of fibroblast in role in fracture healing is not yet fully understood. we proposed this study aiming that fibroblast may be play a special role in fracture healing. Therefore, we discussed this speculation and its possible mechanisms, to find a new therapeutic strategy for fracture healing.

## 2 Characteristics and functions of fibroblasts

### 2.1 The origins and characteristics of fibroblasts

Fibroblasts were first identified in the 1800s by Virchow as “spindle-shaped cells of the connective tissue”. In 1895, Ernst Ziegler termed these cells as “fibroblast” (15). Today, fibroblasts are defined in a number of different ways. Morphologically, fibroblasts typically appear spindle-shaped and elongated, and when activated and differentiate into myofibroblasts, they can spread and become stellate in appearance (16). Though there are some markers considered typical of fibroblasts, such as vimentin and collagen, these are considered nonspecific (12). Fibroblasts possess an impressive plasticity in pathological states where these cells attempt to correct damages and restore tissue homeostasis. Numerous studies now suggest fibroblasts are heterogeneous in their origins, molecular markers, and functions, particularly during the pathological remodeling of organ tissue (17). It appears that distinct niches of fibroblasts engage or are recruited to various tissues in response to injury (12). On top of the different subpopulations of fibroblasts, and the different roles these fibroblasts play as a result, their heterogeneity is also illustrated by the ability of these cells to interconvert with other cell types. In the context of scarring, lung and skin fibroses show transition of adipocytes, fascial fibroblasts, pericytes, and hematopoietic cells into myofibroblasts (18, 19). One established framework classifies fibroblasts on the basis of function during homeostasis, denoting fibroblasts found in various regions of bone, such as the periosteum (the outer membrane of bone), the endosteum (the inner lining of bone), and within the bone marrow. The functions and characteristics of fibroblasts can vary based on their specific location. Bone fibroblasts can exhibit different phenotypes or states. Some may be more quiescent, while others are activated and involved in tissue repair or inflammation (12).

### 2.2 The heterogeneity of fibroblasts

Fibroblasts have been extensively characterized in culture; the advent of single-cell RNA sequencing (scRNA-seq) has recently enabled in-depth exploration of fibroblast identity *in vivo*. Taking advantage of this, numerous studies have described inter- and intra-tissue heterogeneity of fibroblasts across several murine organs (20). scRNA-seq is one of the most widely applied methods used to explore fibroblast heterogeneity. Analysis typically begins by clustering cells in the dataset based on gene expression using computational methods, and annotation of these cluster groups based on cluster gene signatures (21). Fibroblasts from mouse skin, colon, heart, and skeletal muscles show vastly different “matrisomal”(ECM-related) gene expression patterns and share less than 12% of their markers, suggesting that the transcriptional profile of fibroblasts is highly tailored to their specific tissue of residence (22). Fibroblasts are restricted to single tissues and express tissue-specific markers, while universal fibroblasts are dispersed across multiple tissues, show elevated expression of “stemness” genes, and are predicted to serve as progenitors for specialized fibroblasts (23). Fibroblast subtypes are abundantly characterized by the variable expression of specific genes involved in various cellular processes such as extracellular matrix remodeling, cell migration, immune modulation, tissue repair and angiogenesis. This heterogeneity suggests that fibroblasts play multifaceted roles in bone fracture healing, with each subtype contributing to different aspects of the repair process. Regulation of fibrotic fibroblasts by the E26 transformation-specific (ETS) family of transcription factors has also been revealed by scRNAseq analysis of inflamed tissues (21, 24). Recently the researchers demonstrated that expression and the functional effects of exogenous administration of FGFs in fracture repair have been limited to studies of the proto typical family members, however over the past few years molecular technique based on investigate crosstalk among cell types with the development of a scRNA-seq tool such as Cell Chat has given a novel method preserving the spatial integrity of the fibroblasts’ contribution to healing (25).

### 2.3 The function of fibroblasts in health and disease

In homeostasis, fibroblasts are responsible for depositing and maintaining collagen and proteins of the extracellular matrix (ECM), and as previously mentioned, in wound healing, fibroblasts contribute to the formation of new connective tissues by proliferating and secreting collagen (26). These cells are also known to play roles in cancer, angiogenesis, and inflammation (27). In cancer, for example, studies have demonstrated that fibroblasts secrete growth factors that promote tumor cell proliferation. Fibroblasts are also known to secrete cytokines such as Interleukin 8 (IL-8), which are important in inflammation (28, 29). During the process of fracture healing, fibroblasts play a significant role, primarily during the later stages of repair and bone remodeling. It can synthesize and secrete proteins, collagen fibers, elastic fibers, reticular fibers, and organic matrix, all of which play a role in different stages of fracture healing (11).

## 3 The mechanism of fibroblast regulating fracture healing

### 3.1 Cytokines promote osteogenic differentiation of human fibroblasts

**Figure 1:**
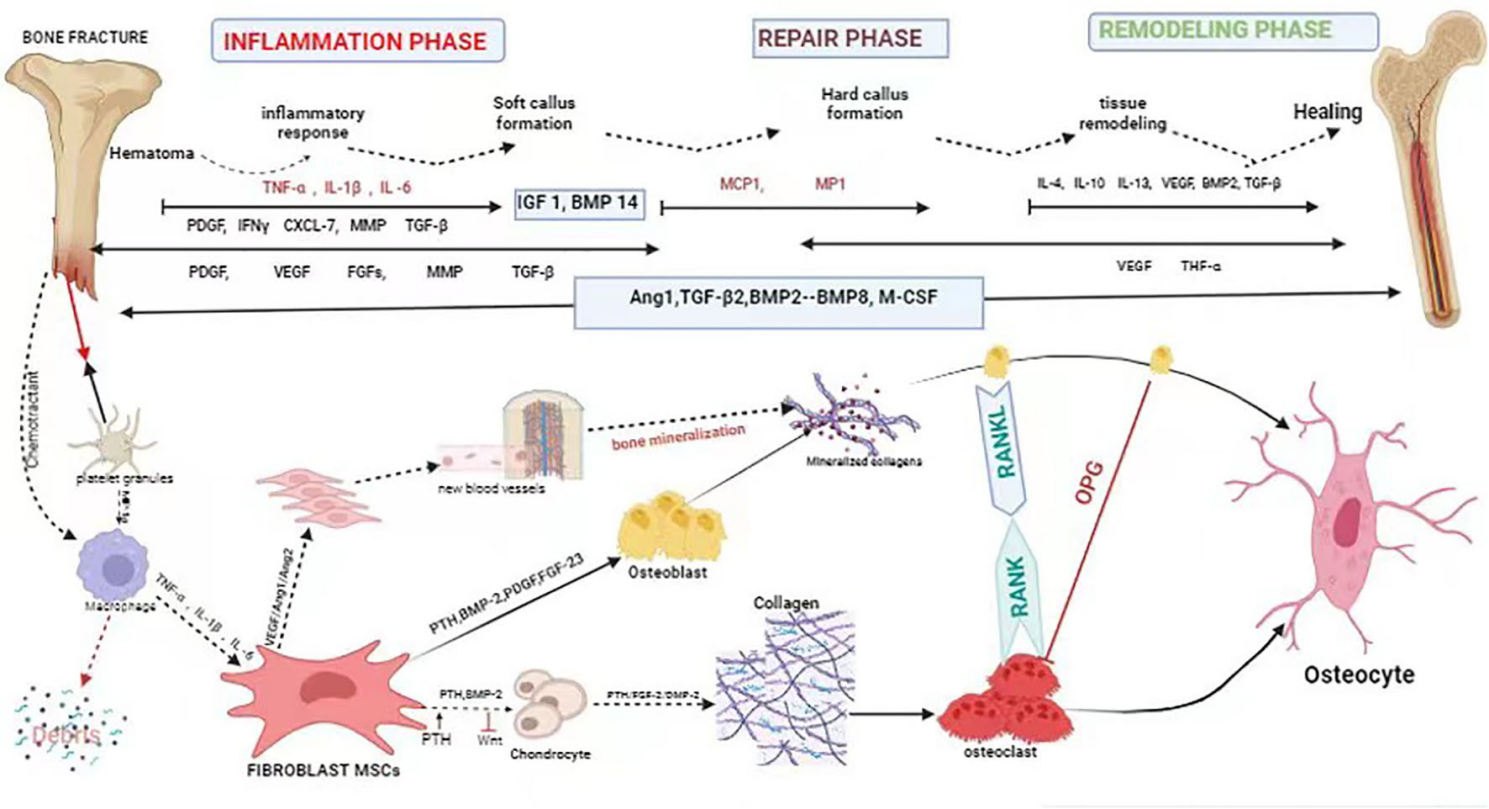
Schematic description of the four phases of fracture healing with sequential need for growth factor. Bone healing can be viewed as a three-stage biological phase (inflammation, repair, and remodelling) which can be further divided into six main sub-steps: hematoma, inflammation, soft callus formation, hard callus formation, remodelling, bone healing. Immune cells are activated and recruited towards the fracture gap. Platelet derived growth factor (PDGF) acts in recruitment and proliferation of mesenchymal stem cells/osteoprogenitor cells. Activation of cytokine, for instance, (VEGF) also paves the way for angiogenesis. Bone morphogenic protein-2 (BMP-2) promotes osteoblastic and chondrocyte differentiation. Fibroblast growth factor-2 (FGF-2) acts mitogenic on MSC/OPC, osteoblasts, chondrocytes and osteoclasts, and enhances matrix synthesis and angiogenesis. Fibroblast growth factor 23 (FGF-23) inhibits osteoblast differentiation and matrix synthesis. IGF, PDGF, and VEGF promote the increase activity of osteoblast proliferation and differentiation. TGFβ has a positive effect to bone remodelling through regulation of both osteoblasts and osteoclasts.

Bone is considered a structurally and functionally complex tissue (30). Fracture healing is a complicated and slow process of repair. It involves systemic or local circumstances, together with some types of cells and growth factors that communicate with adjacent tissues and blood to promote bone healing (31). Fracture healing regenerates bone through a complex series of proliferative and morphogenic events within the injured skeletal tissues. The repair of a bone fracture progresses from the inflammatory stage that follows the initial wound, through intramembranous bone formation, bridging of the fracture gap by chondrogenesis and endochondral bone formation, and finally remodeling of the fracture callus matrix. Each stage of repair requires the communication between diverse tissue types that must be mediated through local cellular growth factors. Although the molecular coordination of fracture repair is largely undefined, examination of the spatial and temporal expression domains has offered indirect evidence for the functional involvement of growth factors and signaling molecules expressed in skeletal development and repair (32). In the process of fracture healing, a variety of growth factors are involved, mainly bone-morphogenetic protein (BMP), transforming growth factor- β (TGF-β), fibroblast growth factors (FGF) and insulin-like growth factor (IGF), etc., which play a role in bone regeneration and repair, cell proliferation and differentiation, as well as the formation of extracellular matrix, which are candidates for therapeutic applications in bone healing (33). Fibroblasts are chemotactic and are attracted to the fracture site by the stress of trauma and by chemokines (e.g., lymphokines, complement, platelet-derived factor, β -transforming growth factor, collagen type I, II, III) (34, 35) (**Figure 1**) (**Table 1**). Thus, fibroblasts play an important regulatory role in fracture healing.

**Table 1:** Summary of growth factors affecting fibroblasts in fracture healing.

GF	Cell source	Biologic effect	Action on fibroblast osteogenic	Reference
**BMP**	Osteoprogenitor cell, osteoblast, Chondro-osteogenesis, Migration of osteoprogenitors, chondrocyte, endothelial cell (BMP-2)	Chondro-osteogenesis, osteo-induction (BMP-2)	regulating bone balance by controlling the differentiation of osteoblasts and osteoclasts BMPs can indirectly influence fibroblast activation and behaviour through their effects on other cell types	(36–38)
**FGF**	Macrophage, monocyte BMSC, chondrocyte, osteoblast, endothelial cell	Angiogenesis, proliferation of fibroblast, and smooth muscle cells of vessels	regulates genes for osteoprogenitor proliferation and differentiation, and apoptosis of osteoblasts.	(39–42)
**IGF**	Osteoblast, chondrocyte hepatocyte, endothelial cell	Regulation of growth, hormone effects	promote the growth and proliferation of various cell types, including fibroblasts involved in tissue repair and bone formation.	(43)
**PDGF**	Platelet, osteoblast endothelial cell, monocyte, macrophage	Proliferation of connective, tissue cells, monocyte/macrophage and smooth muscle cell chemotaxis, angiogenesis	stimulates the proliferation and migration of fibroblasts to the site of injury, including bone fractures.	(44)
**TGF-β**	Platelet, osteoblast, BMSC chondrocyte, endothelial cell, fibroblast, macrophage	Immunosuppression, angiogenesis, stimulation of cell growth, differentiation and ECM synthesis	plays a significant role in promoting fibroblast proliferation and collagen production during tissue repair, bone healing.	(45, 46)
**VEGF**	Osteoblast, platelet	Angiogenesis	Conversion of cartilage into bone, osteoblast proliferation and differentiation, modulates chondrocyte functions and is crucial for proper endochondral ossification	(47, 48)
**CTGF**	Endothelial Cells, Smooth Muscle Cells, Chondrocytes, mesenchymal stem cells	Adhesion, migration, proliferation, and extracellular matrix production	CTGF is often produced by fibroblasts in response to various signalling molecules and mechanical cues, especially during tissue repair and fibrotic processes, are essential processes in tissue repair and osteogenesis.	(49)

#### 3.1.1 FGF

FGF initially was isolated in brain and pituitary extracts long time ago. The mammalian FGF family contains 22 members. Some of them are intracellular FGF (iFGF), which act without binding to FGF receptor (FGFR) (50). In the recent years, the specific structural and functional information on homologous members of this family was determined. FGF are involved in a number of cellular processes that include mitogenesis; chemotaxis, differentiation, and angiogenesis; development of the vascular, nervous, and skeletal systems; maintenance and survival of tissues; and stimulation of wound healing. It plays an important role in the development of vascular, nervous, and skeletal systems promotes the maintenance and survival of certain tissues and stimulates wound healing and tissue repair (51). The FGFR is a highly conserved complex signaling pathway, categorized differently such as canonical (paracrine), hormone-like (endocrine), and intracellular (intracrine). The canonical subfamily comprises 15 known receptor-binding ligands (52, 53). FGF are important signaling molecules that regulate many stages of endochondral bone development. recently various healing of a skeletal fracture, several features of endochondral bone development are reactivated. study on the role of FGF in skeletal fracture healing, evaluated the temporal expression patterns of FGF, FGFR, and molecular markers of bone development over a 14-day period following long bone fracture. These studies identify distinct groups of FGF that are differentially expressed and suggest active stage-specific roles for FGF signaling during the fracture repair process (54). However, FGF and FGFR gene families encode essential signaling molecules that function throughout all stages of endochondral bone development. For example, deficiencies in FGF2 have been linked to decreased bone formation and bone mass (55), while FGF9 and FGF18 signaling regulate hypertrophic chondrocyte differentiation, skeletal vascularization, osteoblast/osteoclast recruitment to the growth plate (56, 57). In addition, FGFR also function in osteogenic differentiation and maturation. FGFR1 signaling in osteo-chondroprogenitor cells regulates osteoblast maturation (58). FGFR2 is important in skeletal growth and bone density (59) and mutations in FGFR2 are linked to a variety of craniosynostosis syndromes in humans (60). FGFR2 has been found to play a key regulatory role in bone development. For example, FGFR2 is involved in the regulation of aqueous extract of Aralia echinocaulis Hand on fracture healing (61). Skeletal overgrowth has been seen in association with FGFR3 deficiency in both mouse and human (62). Disruption of FGF signaling during bone development results in a range of phenotypes from relatively mild shortening of limbs to severe dysmorphology, including truncated and missing limbs.

Acidic FGF (aFGF, FGF1) and basic FGF (bFGF, FGF2) are two typical members of the FGF family (63). In studying the effects of aFGF and ascorbic acid on human periodontal fibroblast growth, osteogenic differentiation, and modulation of the inflammatory response to mechanical stress, it was found that high concentrations of aFGF promoted cell proliferation with short-term stimulation, while prolonged treatment induced the expression of osteogenic markers even at low concentrations (64). Furthermore, it has been demonstrated that bFGF plays an important role in fracture repair (65, 66). bFGF can improve the mechanical stability of fibrous bone scabs and accelerate fracture healing by affecting fibroblast proliferation and collagen synthesis (67). bFGF activates ERK and Akt phosphorylation in a dose dependent manner in both adult and fetal skin fibroblasts, which suggests that bFGF in amniotic fluid plays the most major role in cell proliferation. Application of bFGF from an early fracture healing may lead to better fibroblast proliferation and DNA synthesis through the process of ERK/Akt phosphorylation (68).

#### 3.1.2 BMPs

BMPs were originally discovered to promote bone growth in muscle. They are important morphogens during embryogenesis and development and regulate the maintenance of homeostasis in adult tissues. Further categorized into subgroups based on amino acid similarity: BMP-2/4, BMP-5/6/7/8, BMP-9/10, and BMP-12/13/14. The BMPs belong to the TGF- β superfamily except for BMP-1, which is a metalloprotease. Among them, BMP-2 and BMP-4 play an important role in regulating bone formation (69). In orthopedics, BMPs are naturally secreted multifunctional proteins that play crucial roles throughout the developing skeletal system. BMPs have been proven to be key factors with significant osteogenic functions, regulating bone balance by controlling the differentiation of osteoblasts and osteoclasts (36, 70). Notably, BMP-2 and BMP-7 can significantly enhance osseointegration (37). Therefore, Studies have shown that fibroblasts can differentiate to osteoblasts when induced by BMPs. Dumic-Cule I et al. (71) found that joint fibroblast-like cells can be transformed into osteoblasts under the action of BMP-2 or BMP-2 in combination with TGF-β. The results of Schwarting et al. (72) suggested otherwise. The researchers established an *in vitro* bone-tendon co-culture model in mice designed to test the region-specific effects of BMP-2 on osteoblast and fibroblast differentiation. The expression of osteogenic markers in the osteoblast region was significantly upregulated by BMP-2 stimulation, whereas the effect on fibroblasts was not significant.

Fibroblasts showed similar osteogenic responses to BMP-4 and BMP-2/7, both of which significantly increased ALP activity and calcium production (73). Simanshu DK’s experimental results proved that tumor necrosis factor-α could change the collagen phenotype of fibroblasts from type I and type III to predominantly secreting type I collagen, and induced fibroblasts to express Ras and BMP type I receptors. Type I collagen stimulates the formation of “bone nodule”-like structures, forming the bone microenvironment. Ras protein, through the activation of adenylate cyclase and its own guanine nucleosidase activity, causes the guanine nucleotide conversion, which leads to the activation of mitogen-kinase, resulting in the phosphorylation of nuclear transcription factors and the activation of cell division, proliferation and development, and differentiation; and the BMP I receptor may be the target of the action of BMP2, thereby inducing the fibroblasts, which show multidirectional potential for differentiation under the action of TNF-a, to differentiate towards the direction of osteoclasts (74). In addition, TNF-α combined with BMP-2 induces fibroblasts to express the osteoblast-specific transcription factors Cbfa1 and osteocalcin mRNA and regulates osteoblast differentiation (75). Another study found that BMP-2 acts synergistically with TNF to stimulate the production of nerve growth factor in fibroblasts through an indirect mechanism and plays an important role in the regulation of peripheral nerve regeneration after fracture injury (76). Yu et al. (77) found that BMP-9 could induce fibroblasts to differentiate into hyaline cartilage, and then carried out *in vivo* experiments to implant hyaline cartilage into acutely damaged joints. It was found that the transplanted hyaline cartilage survived and maintained the hyaline cartilage phenotype after implantation, but did not form mature articular cartilage. Thus, fibroblasts could also be a cellular source of articular cartilage. *In vivo* experiments are different from *in vitro* experiments and may need to take the influencing factors of the body’s immune microenvironment into account, and the specific mechanisms still need to be further explored.

#### 3.1.3 TGF-β

TGF-β stimulates chondrocyte proliferation and matrix synthesis. TGF-β expression has been identified in the bone and cartilage of fracture callus by several investigators, suggesting an autocrine or paracrine role in this process (45, 46). Together with activin, inhibin, Mullerian inhibiting substance and BMPs, TGF-β forms the TGF-β superfamily. A variety of cells in the body can secrete TGF-β in an inactive state, with three isoforms, TGF-β1, TGF-β2, and TGF-β3. Under acidic conditions, especially near fractures and wounds, TGF-β can be activated into a multifunctional active peptide, with the main function of regulating cell growth and differentiation, ECM production, and immune modulation (78). In 2014, Aloise et al. (79) addressed the role of TGF-β1 on the induction of osteogenic differentiation in human dermal fibroblasts, and the analysis revealed that the addition of TGF-β1 to the osteogenic medium increased the activity of ALP and the amount of osteocalcin in the supernatant of fibroblasts, but that TGF-β1 did not alter the presence of mineralized calcium phosphate deposits in fibroblasts. Yamamoto et al. (80) induced human fibroblasts to display an osteoblast phenotype, i.e., transformed osteoblasts, by culturing them with the TGF-β receptor inhibitor ALK5i II. The expression profile of osteogenic-related genes in transformed osteoblasts was similar to that of primary human osteoblasts, and the addition of vitamin D3 to ALK5i II induced additional osteoblast-like traits with a transformation efficiency of approximately 90%. In addition, the transformed osteoblasts produce a large amount of calcified bone matrix, similar to mesenchymal cells-induced osteoblasts, and transplantation of them into artificially induced femoral defect lesions in immunodeficient mice promotes bone healing.

#### 3.1.4 IGFs

IGFs are a class of multifunctional cell proliferation regulators that play an important role in promoting cell differentiation, proliferation, and individual growth and development (81). They are mainly of two types, IGF-1 and IGF-2. IGF-1 plays many important roles in both bone development and remodeling. IGF-1 is the most abundant growth factor stored in bone, and with its receptor IGF1R forms a major growth-promoting signaling system for the skeleton (82). IGF-1 was found to promote the proliferation of fibroblasts during osteogenesis as well as increase their osteogenic capacity (83). Two concentrations (10 μM and 100 μM) of L-carnitine were added to fibroblasts, after which fibroblast osteoblast differentiation was reassessed. The results showed that 100 μM L-carnitine inhibited fibroblast differentiation as evidenced by decreased ALP activity, mineralized nodule formation, calcium deposition, and down-regulation of the osteogenic marker genes ALP, Runx2, and OCN, while 10 μM L-carnitine exerted the opposite effect. Mechanistically, the IGF-1/PI3K/Akt signaling pathway plays an important role in this (83). A report demonstrated the trans differentiation of human fibroblasts into functional osteoblasts using insulin-like growth factor binding protein 7(IGFBP7). Cytological experiments confirmed that recombinant IGFBP7 induced a phenotypic switch from fibroblasts to osteoblasts. *In vivo* experiments revealed that when 1 μg/mL IGFBP7-treated fibroblasts were cultured for 14 days and then transplanted into mice, several samples from the treatment group showed substantial bone formation and their mean BV values were significantly higher, as well as the appearance of mineralized tissues, and nodularity was also observed in micro-CT images. The main mechanism lies in the fact that IGFBP7 triggers an IL-6-dependent pathway in osteoblast reprogramming in fibroblasts and correlates with cellular senescence, which is a new strategy for bone regeneration (84).

#### 3.1.5 Other factors

It is well known that human skin fibroblasts (HSFs) have a multidirectional differentiation potential close to that of mesenchymal stem cells. A study on the mechanism of osteogenic differentiation of HSFs found that depolymerization of microfilaments inhibited the expression of osteogenesis-related proteins and ALP activity of HSFs, whereas polymerization of microfilaments enhanced the osteogenic differentiation of HSFs. PDLIM5, a cytoskeleton-associated protein, can mediate osteogenic differentiation of fibroblasts by affecting microfilament formation and polymerization (85). Another study found that membrane-bound protein A2 promotes the ossification of ligament fibroblasts in patients with ankylosing spondylitis, and experiments suggest that it may function through the extracellular signaling-related kinase pathway (86). In addition, pretreatment with parathyroid hormone combined with mechanical distraction also promotes periodontal fibroblast osteogenesis (87). Osteogenic factors are commonly used in orthopedics to promote bone growth and improve fracture healing. Osteogenic oxysterols are naturally occurring molecules that have been shown to induce osteogenic differentiation *in vitro* and promote spinal fusion *in vivo*. Among more than 100 synthesized oxysterol analogs, Oxy133 induced significant expression of the osteogenic markers Runx2, Osterix, ALP, bone salivary proteins, and osteocalcin in mouse embryonic fibroblasts, C3H10T1/2, and mouse bone marrow stromal cells, M2-10B4.Oxy133 has great potential as an osteogenic molecule, and small molecule osteogenic oxysterols could be used for therapeutic development as next-generation osteoanabolic agent (88).

### 3.2 Effect of mechanical stress on osteogenic differentiation of fibroblasts

Distraction osteogenesis is a well-recognized clinical treatment for limb length discrepancies and skeletal deformities. Conditioned media from mesenchymal stromal cells and periodontal fibroblasts stimulated by cyclic stretching were found to promote bone healing of cranial cap defects in mice (89). In addition, numerous studies have confirmed that tension at the distributed action gap correlates with plasma bone-specific ALP activity during distraction, as well as the regulatory role of TGF-β1 on ALP activity during fracture healing (90, 91). Based on this, Yeung et al. (92) conducted a study on TGF-β1 and mechanical force. The results of the study showed that mechanical forces induced and maintained TGF-β1 expression in osteoblasts and fibroblast-like cells of distraction healing tissues, and in turn, TGF-β1 functioned in transducing mechanical stimuli into biological tissues during distraction osteogenesis. Connexin43 (Cx43) is a gap junction protein that plays a role in bone formation, maintains endosteal homeostasis and regulates bone remodeling (93). Yang et al. (94) observed that gene expression of OCN, ALP, COLI and Cx43 proteins was significantly upregulated in fibroblasts under mechanical stress. In contrast, Li et al. (95) found that cyclic mechanical tension increased Cx43 expression in human fibroblasts and further up- regulated osteogenic (e.g., Runx2, Osterix, OPG) and down-regulated osteoclast (e.g., RANKL) generating signaling molecules, with a mechanism of action that may be involved in osteoblastic or osteoclastogenic differentiation potentials of human fibroblasts through the Cx43-ERK1/2 signaling pathway. In conclusion, mechanical stress can influence the osteogenic transformation of fibroblasts, thereby promoting fracture healing.

### 3.3 Bone microenvironment controls functions of pre-osteoblasts and fibroblasts

Fibroblasts and osteoblasts secret matrix to replace granulation tissue and play a crucial part in achieving bone regeneration. In the repair phase, fibroblasts are recruited and activated for a fibrotic response to generate a transient collagenous matrix with enhanced mechanical strength, to replace the granulation tissue (96). Failure to terminate the fibrotic response in pathological conditions, such as polytrauma of bone and skeletal muscle, induces pathological scarring, termed fibrosis, leading to excess collagen deposition (97, 98). The accumulation of fibrous tissue within the fracture callus interferes with bone defect consolidation (98). After the fibrotic response, endochondral ossification occurs in the inter-cortical and cancellous bone areas, while intramembranous ossification occurs in the subperiosteal area and the adjacent soft tissue areas (99). In the inter-cortical and cancellous bone areas, MSCs proliferate and further differentiate into chondrocytes, which secrete cartilage matrix that transforms the fiber-rich granulation tissue into a soft callus with higher stiffness (96). Then, osteoblasts secrete type I collagen and participate in bone mineralization, transforming soft callus into hard callus (100). In the subperiosteal area and the adjacent soft tissue areas, pre- osteoblasts and MSCs are recruited. These cells differentiate into osteoblasts, which secrete extracellular matrix proteins and generate bone mineral crystals, and directly form the hard callus under the periosteum (4). Then, the new bone is remodeled by osteoblasts, osteoclasts and other cells, to restore the initial structure of bone in addition, scaffolds were manufactured to promote bone regeneration by regulating cell behavior. For example, polyether ether ketone (PEEK)/titanium dioxide (TiO2) scaffold coating hydroxyapatite (HA) demonstrated a strong potential to support new bone regeneration via promoting pre-osteoblast adhesion, proliferation and osteoblastic differentiation (101).

## 4 Novel promising strategy of fibroblasts to osteoblasts

Bone formation (bone ossification or osteogenesis) involves 2 distinct mechanisms: endochondral ossification (EO) and intramembranous ossification (IO) (102). EO begins with the transformation of mesenchymal tissue into a cartilage intermediate, which is later mineralized to form the bone of the axial skeleton and long bones (103). IO, in contrast, is a process where bone tissue forms without the involvement of cartilage precursors. IO primarily occurs in flat bones of the skull (such as the parietal and frontal bones) and other bones like the clavicles. Like mentioned age can involve in impairment of bone healing and bone regeneration. Age-related heterogeneity of fibroblasts can impact fracture healing by affecting their proliferation, secretory profile, collagen production, angiogenic capabilities, and interactions with other cell types involved in bone repair. Most elderly people develop osteoporosis with age. Osteoporosis results in bone loss and an increased risk of fractures that can lead to death (104). Several techniques for promoting bone regeneration and repair have been developed to reduce bone fracture-related mortality in elderly individuals. These techniques include allografts (105), gene therapy and cell-based therapy, all of which have undergone testing in patients (106). Recent studies have highlighted that somatic cells can readily be converted into specific cell types without the involvement of a pluripotent state (107). This reprogramming can directly induce the formation of the intended cell type from somatic cells, whereas it can also be used to safely generate substantial amounts of the desired cell type (108). Thus, there have been a number of attempts in recent years to treat several diseases using direct cell reprogramming techniques (109). Direct reprogramming is one of the currently techniques for enhancing bone healing by converting patient fibroblasts directly to osteoblast-like cells, bypassing the necessity for the cells to transition through a stem cell phase (21, 110, 111).

Osteoblasts produce calcified bone matrix and contribute to bone formation and remodeling, rejuvenating their function could potentially enhance bone regeneration. by converting fibroblasts into osteoblasts, if this can be achieved, then a sufficient number of osteoblasts may be obtained from the somatic cells of a patient with osteolytic disease and issues associated with bone healing. Since osteoblasts play a central role in bone formation, direct reprogramming of fibroblasts into osteoblasts may be a new approach to treating fractures in the elderly. Recently, it was reported that forced expression of four transcription factors, Runt-related transcription factor 2 (Runx2), Osterix (Osx), Octamer-binding transcription factor 3/4 (Oct4), and L-Myc, can directly convert human fibroblasts to functional osteoblasts as assayed by gene expression and mineralization. Other combinations, including Oct6, Oct9, or N-myc, can also convert human fibroblasts to osteoblasts (108). However, osteoblast gene expression and mineralization *in vitro* are not sufficient assays of osteoblast characterization as even the presence of only a small fraction of osteoblast lineage cells can yield positive results. In 2015, Yamamoto’s group proposed that transduction of Oct9 and N-myc could transform human fibroblasts into osteoblast-like cells, thereby inducing an osteoblast-like phenotype and expression of Runx2 and osteocalcin (112). Yamamoto et al. (113) also constructed a plasmid vector encoding Oct4, Osterix, and L-Myc that was transfected into normal human fibroblasts cultured in osteogenic medium. The expression of bone matrix-producing and osteoblast-specific genes demonstrated that human fibroblasts could be directly transformed into osteoblasts; calcitonoid deposits were formed *in situ* after transplantation of the cells into mice. These results strongly suggest that plasmid-induced osteoblasts are a new cell-based therapy for bone disease. In addition, retroviral gene transfection of the osteoblast transcription factor Runx2/Cbfa1 also promotes osteogenic differentiation of primary dermal fibroblasts cultured in monolayer (114). Murine embryonic fibroblasts reprogrammed with transcription factors (c-Myc and Oct4) and hLMP-3 were transplanted into an animal model of femoral defects in rats, which effectively promoted the healing time and efficiency of bone defects (11). This shows that reprogramming is an effective treatment technique in the field of orthopedics.

Several previous studies have demonstrated that fibroblasts applied to bone tissue engineering have also achieved good results in promoting bone regeneration. Sommar et al. (115) combined human dermal fibroblasts with microporous gelatin microcarriers to form a three-dimensional osteogenic biomaterial, which produces a mineralized extracellular matrix *in vitro* when exposed to osteogenic induction medium. Human dermal fibroblasts transplanted on gelatin microcarriers for osteogenic induction were subsequently implanted into rats with femoral defects, with the aim of finding out whether the fibroblasts retained their osteogenic-induced phenotype *in vivo*. CT 4 weeks after transplantation showed that the cells survived and maintained their osteogenic properties, and although the defects did not heal at 4 weeks, the stability of the phenotype of the cells after *in vitro* induction has been confirmed, suggesting their possible value in new protocols for bone regeneration.

## 5 Conclusion

A variety of growth factors can promote the osteogenesis of fibroblasts during fracture healing and play a role in bone regeneration and repair, cell proliferation and differentiation, and extracellular matrix formation. The application of genetic coding technology. Fibroblasts has a high proliferative capacity, which does not decline with increasing donor age. Thus, direct reprogramming may provide another option for such patients in whom MSC transplantation might not be an appropriate therapy, fibroblasts will also be a proven therapeutic approach in the field of orthopedics. In addition, physical effects, such as mechanical stress, can also promote the transformation of fibroblasts into osteoblasts and thus promote fracture healing. This information is critical for strategies to promote bone regeneration, and translational clinical applications targeting fibroblasts will be a boon to fracture patients.

## Author contributions

HuW: Writing – original draft. LQ: Writing – original draft. CS: Writing – original draft. KJ: Writing – original draft. PR: Writing – original draft. HeW: Writing – original draft, Writing – review & editing. LW: Writing – original draft, Writing – review & editing.

## References

[1] Du MJ Lin YH Chen WT Zhao H. Advances in the application of ultrasound for fracture diagnosis and treatment. Eur Rev Med Pharmacol Sci. (2022) 26:7949–54. doi: 10.26355/eurrev_202211_30146 36394743

[2] Global, regional, and national burden of bone fractures in 204 countries and territories, 1990-2019: a systematic analysis from the Global Burden of Disease Study 2019. Lancet Healthy Longevity. (2021) 2:e580–e92. doi: 10.1016/S2666-7568(21)00172-0 PMC8547262 34723233

[3] Saul D Khosla S. Fracture healing in the setting of endocrine diseases, aging, and cellular senescence. Endocrine Rev. (2022) 43:984–1002. doi: 10.1210/endrev/bnac008 35182420 PMC9695115

[4] Guerquin MJ Duquenne C Coffigny H Rouiller-Fabre V Lambrot R Bakalska M. Sex-specific differences in fetal germ cell apoptosis induced by ionizing radiation. Hum Reprod (Oxford England). (2009) 24:670–8. 10.1093/humrep/den410 19088112

[5] Johnell O Kanis JA. An estimate of the worldwide prevalence and disability associated with osteoporotic fractures. Osteoporosis Int J established as result cooperation between Eur Foundation Osteoporosis Natl Osteoporosis Foundation USA. (2006) 17:1726–33. doi: 10.1007/s00198-006-0172-4 16983459

[6] Claes L Recknagel S Ignatius A. Fracture healing under healthy and inflammatory conditions. Nat Rev Rheumatol. (2012) 8:133–43. doi: 10.1038/nrrheum.2012.1 22293759

[7] Maruyama M Rhee C Utsunomiya T. Modulation of the inflammatory response and bone healing. Front endocrinology. (2020) 11:386. doi: 10.3389/fendo.2020.00386 PMC7325942 32655495

[8] Arron JR Choi Y. Bone versus immune system. Nature. (2000) 408:535–6. doi: 10.1038/35046196 11117729

[9] Lee B Lee SH Shin K. Crosstalk between fibroblasts and T cells in immune networks. Front Immunol. (2022) 13:1103823. doi: 10.3389/fimmu.2022.1103823 36700220 PMC9868862

[10] Parsonage G Falciani F Burman A Filer A Ross E Bofill M. Global gene expression profiles in fibroblasts from synovial, skin and lymphoid tissue reveals distinct cytokine and chemokine expression patterns. Thromb haemostasis. (2003) 90:688–97. doi: 10.1160/TH03-04-0208 14515190

[11] Driskell RR Lichtenberger BM Hoste E Kretzschmar K Simons BD Charalambous M. Distinct fibroblast lineages determine dermal architecture in skin development and repair. Nature. (2013) 504:277–81. doi: 10.1038/nature12783 PMC3868929 24336287

[12] Lynch MD Watt FM. Fibroblast heterogeneity: implications for human disease. J Clin Invest. (2018) 128:26–35. doi: 10.1172/JCI93555 29293096 PMC5749540

[13] Mascharak S desJardins-Park HE Davitt MF Griffin M Borrelli MR Moore AL. Preventing Engrailed-1 activation in fibroblasts yields wound regeneration without scarring. Sci (New York N.Y.). (2021) 372(6540):eaba2374. doi: 10.1126/science.aba2374 PMC9008875 33888614

[14] Correa-Gallegos D Jiang D Rinkevich Y. Fibroblasts as confederates of the immune system. Immunol Rev. (2021) 302:147–62. doi: 10.1111/imr.12972 34036608

[15] Plikus MV Wang X Sinha S Forte E Thompson SM Herzog EL. Fibroblasts: Origins, definitions, and functions in health and disease. Cell. (2021) 184:3852–72. doi: 10.1016/j.cell.2021.06.024 PMC8566693 34297930

[16] Ravikanth M Soujanya P Manjunath K Saraswathi TR Ramachandran CR. Heterogenecity of fibroblasts. J Oral Maxillofac Pathol. (2011) 15:247–50. doi: 10.4103/0973-029X.84516 PMC3329689 22529592

[17] Kalluri R. The biology and function of fibroblasts in cancer. Nat Rev Cancer. (2016) 16:582–98. doi: 10.1038/nrc.2016.73 27550820

[18] Weiskirchen R Weiskirchen S Tacke F. Organ and tissue fibrosis: Molecular signals, cellular mechanisms and translational implications. Mol aspects Med. (2019) 65:2–15. doi: 10.1016/j.mam.2018.06.003 29958900

[19] Shook BA Wasko RR Mano O Rutenberg-Schoenberg M Rudolph MC Zirak B. Dermal adipocyte lipolysis and myofibroblast conversion are required for efficient skin repair. Cell Stem Cell. (2020) 26:880–95.e6. doi: 10.1016/j.stem.2020.03.013 32302523 PMC7853423

[20] Xie T Wang Y Deng N Huang G Taghavifar F Geng Y. Single-cell deconvolution of fibroblast heterogeneity in mouse pulmonary fibrosis. Cell Rep. (2018) 22:3625–40. doi: 10.1016/j.celrep.2018.03.010 PMC5908225 29590628

[21] Ascensión AM Fuertes-Álvarez S Ibañez-Solé O Izeta A Araúzo-Bravo MJ. Human dermal fibroblast subpopulations are conserved across single-cell RNA sequencing studies. J Invest Dermatol. (2021) 141:1735–44.e35. doi: 10.1016/j.jid.2020.11.028 33385399

[22] Muhl L Genové G Leptidis S Liu J He L Mocci G. Single-cell analysis uncovers fibroblast heterogeneity and criteria for fibroblast and mural cell identification and discrimination. Nat Commun. (2020) 11:3953. doi: 10.1038/s41467-020-17740-1 32769974 PMC7414220

[23] Buechler MB Pradhan RN Krishnamurty AT Cox C Calviello AK Wang AW. Cross-tissue organization of the fibroblast lineage. Nature. (2021) 593:575–9. doi: 10.1038/s41586-021-03549-5 33981032

[24] Yan M Komatsu N Muro R Huynh NC Tomofuji Y Okada Y. ETS1 governs pathological tissue-remodeling programs in disease-associated fibroblasts. Nat Immunol. (2022) 23:1330–41. doi: 10.1038/s41590-022-01285-0 35999392

[25] Jin S Guerrero-Juarez CF Zhang L. Inference and analysis of cell-cell communication using CellChat. Nat Commun. (2021) 12:1088. doi: 10.1038/s41467-021-21246-9 33597522 PMC7889871

[26] Kallis PJ Friedman AJ. Collagen powder in wound healing. J Drugs Dermatol JDD. (2018) 17:403–8. 29601617

[27] Kendall RT Feghali-Bostwick CA. Fibroblasts in fibrosis: novel roles and mediators. Front Pharmacol. (2014) 5:123. doi: 10.3389/fphar.2014.00123 24904424 PMC4034148

[28] Biffi G Tuveson DA. Diversity and biology of cancer-associated fibroblasts. Physiol Rev. (2021) 101:147–76. doi: 10.1152/physrev.00048.2019 PMC7864232 32466724

[29] Mezawa Y Orimo A. Phenotypic heterogeneity, stability and plasticity in tumor-promoting carcinoma-associated fibroblasts. FEBS J. (2022) 289:2429–47. doi: 10.1111/febs.15851 33786982

[30] Dimitriou R Jones E McGonagle D Giannoudis PV. Bone regeneration: current concepts and future directions. BMC Med. (2011) 9:66. doi: 10.1186/1741-7015-9-66 21627784 PMC3123714

[31] Einhorn TA Gerstenfeld LC. Fracture healing: mechanisms and interventions. Nat Rev Rheumatol. (2015) 11:45–54. doi: 10.1038/nrrheum.2014.164 25266456 PMC4464690

[32] Wang IE Shan J Choi R Oh S Kepler CK Chen FH. Role of osteoblast-fibroblast interactions in the formation of the ligament-to-bone interface. J Orthop Res. (2007) 25:1609–20. doi: 10.1002/jor.20475 17676622

[33] Li X Wang C Xiao J McKeehan WL Wang F. Fibroblast growth factors, old kids on the new block. Semin Cell Dev Biol. (2016) 53:155–67. doi: 10.1016/j.semcdb.2015.12.014 PMC4875805 26768548

[34] Meng GL Hu YY Pu Q Lu R Yang L Wang J. Reciprocal action between BMP-2 and BMP-3 in cultured fibroblast *in vitro* . Chin J traumatology = Zhonghua chuang shang za zhi. (2003) 6:3–7. 12542956

[35] Bi JH Zhu TY Shi YY. Osteogenesis ability of fibroblasts. J ClinicalRehabilitative Tissue Eng Res. (2007) 11:2733–6.

[36] Poynton AR Lane JM. Safety profile for the clinical use of bone morphogenetic proteins in the spine. Spine. (2002) 27:S40–8. doi: 10.1097/00007632-200208151-00010 12205419

[37] Lavery K Swain P Falb D Alaoui-Ismaili MH. BMP-2/4 and BMP-6/7 differentially utilize cell surface receptors to induce osteoblastic differentiation of human bone marrow-derived mesenchymal stem cells. J Biol Chem. (2008) 283:20948–58. doi: 10.1074/jbc.M800850200 PMC3258927 18436533

[38] Liu DD Zhang CY Liu Y Li J Wang YX Zheng SG. RUNX2 regulates osteoblast differentiation via the BMP4 signaling pathway. J Dental Res. (2022) 101:1227–37. doi: 10.1177/00220345221093518 35619284

[39] Hankemeier S Keus M Zeichen J Jagodzinski M Barkhausen T Bosch U. Modulation of proliferation and differentiation of human bone marrow stromal cells by fibroblast growth factor 2: potential implications for tissue engineering of tendons and ligaments. Tissue engineering. (2005) 11:41–9. doi: 10.1089/ten.2005.11.41 15738660

[40] Shu C Smith SM Little CB Melrose J. Use of FGF-2 and FGF-18 to direct bone marrow stromal stem cells to chondrogenic and osteogenic lineages. Future Sci OA. (2016) 2:Fso142. doi: 10.4155/fsoa-2016-0034 PMC5242207 28116125

[41] Cha HJ Yun JI Han NR Kim HY Baek S Lee SH. Generation of embryonic stem-like cells from *in vivo*-derived porcine blastocysts at a low concentration of basic fibroblast growth factor. Reprod Domest Anim = Zuchthygiene. (2018) 53:176–85. doi: 10.1111/rda.13088 29110378

[42] Marie PJ. Fibroblast growth factor signaling controlling bone formation: an update. Gene. (2012) 498:1–4. doi: 10.1016/j.gene.2012.01.086 22342254

[43] Locatelli V Bianchi VE. Effect of GH/IGF-1 on bone metabolism and osteoporsosis. Int J endocrinology. (2014) 2014:235060. doi: 10.1155/2014/235060 25147565 PMC4132406

[44] Andrae J Gallini R Betsholtz C. Role of platelet-derived growth factors in physiology and medicine. Genes Dev. (2008) 22:1276–312. doi: 10.1101/gad.1653708 PMC2732412 18483217

[45] Rosier RN O'Keefe RJ Hicks DG. The potential role of transforming growth factor beta in fracture healing. Clin orthopaedics related Res. (1998) (355 Suppl):S294–300. doi: 10.1097/00003086-199810001-00030 9917649

[46] Liu J Zhang J Lin X Boyce BF Zhang H Xing L. Age-associated callus senescent cells produce TGF-β1 that inhibits fracture healing in aged mice. J Clin Invest. (2022) 132(8):e148073. doi: 10.1172/JCI148073 35426372 PMC9012290

[47] Liu Y Olsen BR. Distinct VEGF functions during bone development and homeostasis. Archivum immunologiae therapiae experimentalis. (2014) 62:363–8. doi: 10.1007/s00005-014-0285-y 24699630

[48] Hu K Olsen BR. The roles of vascular endothelial growth factor in bone repair and regeneration. Bone. (2016) 91:30–8. doi: 10.1016/j.bone.2016.06.013 PMC4996701 27353702

[49] Leask A Abraham DJ. The role of connective tissue growth factor, a multifunctional matricellular protein, in fibroblast biology. Biochem Cell Biol = Biochimie biologie cellulaire. (2003) 81:355–63. doi: 10.1139/o03-069 14663501

[50] Itoh N Ornitz DM. Fibroblast growth factors: from molecular evolution to roles in development, metabolism and disease. J Biochem. (2011) 149:121–30. doi: 10.1093/jb/mvq121 PMC3106964 20940169

[51] Beenken A Mohammadi M. The FGF family: biology, pathophysiology and therapy. Nat Rev Drug discovery. (2009) 8:235–53. doi: 10.1038/nrd2792 PMC3684054 19247306

[52] Itoh N. Hormone-like (endocrine) Fgfs: their evolutionary history and roles in development, metabolism, and disease. Cell Tissue Res. (2010) 342:1–11. doi: 10.1007/s00441-010-1024-2 20730630 PMC2948652

[53] Du X Xie Y Xian CJ Chen L. Role of FGFs/FGFRs in skeletal development and bone regeneration. J Cell Physiol. (2012) 227:3731–43. doi: 10.1002/jcp.24083 22378383

[54] Schmid GJ Kobayashi C Sandell LJ Ornitz DM. Fibroblast growth factor expression during skeletal fracture healing in mice. Dev dynamics an Off Publ Am Assoc Anatomists. (2009) 238:766–74. doi: 10.1002/dvdy.21882 PMC2688661 19235733

[55] Montero A Okada Y Tomita M Ito M Tsurukami H Nakamura T. Disruption of the fibroblast growth factor-2 gene results in decreased bone mass and bone formation. J Clin Invest. (2000) 105:1085–93. doi: 10.1172/JCI8641 PMC300831 10772653

[56] Liu Z Lavine KJ Hung IH Ornitz DM. FGF18 is required for early chondrocyte proliferation, hypertrophy and vascular invasion of the growth plate. Dev Biol. (2007) 302:80–91. doi: 10.1016/j.ydbio.2006.08.071 17014841

[57] Hung IH Yu K Lavine KJ Ornitz DM. FGF9 regulates early hypertrophic chondrocyte differentiation and skeletal vascularization in the developing stylopod. Dev Biol. (2007) 307:300–13. doi: 10.1016/j.ydbio.2007.04.048 PMC2267922 17544391

[58] Jacob AL Smith C Partanen J Ornitz DM. Fibroblast growth factor receptor 1 signaling in the osteo-chondrogenic cell lineage regulates sequential steps of osteoblast maturation. Dev Biol. (2006) 296:315–28. doi: 10.1016/j.ydbio.2006.05.031 PMC2077084 16815385

[59] Yu K Xu J Liu Z Sosic D Shao J Olson EN. Conditional inactivation of FGF receptor 2 reveals an essential role for FGF signaling in the regulation of osteoblast function and bone growth. Dev (Cambridge England). (2003) 130:3063–74. doi: 10.1242/dev.00491 12756187

[60] Marie PJ Kaabeche K Guenou H. Roles of FGFR2 and twist in human craniosynostosis: insights from genetic mutations in cranial osteoblasts. Front Oral Biol. (2008) 12:144–59. doi: 10.1159/000115036 18391499

[61] Yin X Li L Zheng LL Zhang WQ Zhu J Pei LP. [Influence of aqueous extract of Aralia echinocaulis Hand.-Mazz on the expression of fracture healing-related factor receptors]. Zhongguo gu shang = China J orthopaedics traumatology. (2011) 24:761–5. 22007587

[62] Toydemir RM Brassington AE Bayrak-Toydemir P Krakowiak PA Jorde LB Whitby FG. A novel mutation in FGFR3 causes camptodactyly, tall stature, and hearing loss (CATSHL) syndrome. Am J Hum Genet. (2006) 79:935–41. doi: 10.1086/508433 PMC1698566 17033969

[63] Wang J Liu S Li J Yi Z. The role of the fibroblast growth factor family in bone-related diseases. Chem Biol Drug design. (2019) 94:1740–9. doi: 10.1111/cbdd.13588 31260189

[64] Knaup I Symmank J Bastian A Neuss S Pufe T Jacobs C. Impact of FGF1 on human periodontal ligament fibroblast growth, osteogenic differentiation and inflammatory reaction *in vitro* . J Orofac Orthop. (2022) 83:42–55. doi: 10.1007/s00056-021-00363-6 34874457

[65] Sekiguchi H Uchida K Matsushita O Inoue G Nishi N Masuda R. Basic fibroblast growth factor fused with tandem collagen-binding domains from clostridium histolyticum collagenase colG increases bone formation. BioMed Res Int. (2018) 2018:8393194. doi: 10.1155/2018/8393194 29770338 PMC5889866

[66] Zhang H Kot A Lay YE Fierro FA Chen H Lane NE. Acceleration of fracture healing by overexpression of basic fibroblast growth factor in the mesenchymal stromal cells. Stem Cells Transl Med. (2017) 6:1880–93. doi: 10.1002/sctm.17-0039 PMC6430058 28792122

[67] Pacicca DM Patel N Lee C Salisbury K Lehmann W Carvalho R. Expression of angiogenic factors during distraction osteogenesis. Bone. (2003) 33:889–98. doi: 10.1016/j.bone.2003.06.002 14678848

[68] Akita S Akino K Hirano A. Basic fibroblast growth factor in scarless wound healing. Adv Wound Care. (2013) 2:44–9. doi: 10.1089/wound.2011.0324 PMC3623580 24527324

[69] Begam H Nandi SK Kundu B Chanda A. Strategies for delivering bone morphogenetic protein for bone healing. Materials Sci engineering. C Materials Biol applications. (2017) 70:856–69. doi: 10.1016/j.msec.2016.09.074 27770964

[70] Lissenberg-Thunnissen SN de Gorter DJ Sier CF Schipper IB. Use and efficacy of bone morphogenetic proteins in fracture healing. Int orthopaedics. (2011) 35:1271–80. doi: 10.1007/s00264-011-1301-z PMC3167450 21698428

[71] Dumic-Cule I Peric M Kucko L Grgurevic L Pecina M Vukicevic S. Bone morphogenetic proteins in fracture repair. Int Orthop. (2018) 42:2619–26. doi: 10.1007/s00264-018-4153-y 30219967

[72] Schwarting T Schenk D Frink M Benölken M Steindor F Oswald M. Stimulation with bone morphogenetic protein-2 (BMP-2) enhances bone-tendon integration *in vitro* . Connect Tissue Res. (2016) 57:99–112. doi: 10.3109/03008207.2015.1087516 26558768

[73] Myllyla RM Haapasaari KM Lehenkari P Tuukkanen J. Bone morphogenetic proteins 4 and 2/7 induce osteogenic differentiation of mouse skin derived fibroblast and dermal papilla cells. Cell Tissue Res. (2014) 355:463–70. doi: 10.1007/s00441-013-1745-0 24253465

[74] Simanshu DK Nissley DV McCormick F. RAS proteins and their regulators in human disease. Cell. (2017) 170:17–33. doi: 10.1016/j.cell.2017.06.009 28666118 PMC5555610

[75] Deng L Feng W Zhang Y Zhu Y. Expression of core-binding factor a1 by human skin fibroblasts induced *in vitro* . Zhonghua wai ke za zhi [Chinese J surgery]. (2002) 40:592–5. 12417072

[76] Hattorl A Tsujimoto M Hayashi K Kohno M. Bone morphogenetic protein-2 is markedly synergistic with tumor necrosis factor in stimulating the production of nerve growth factor in fibroblasts. Biochem Mol Biol Int. (1996) 38:1095–101. 8739030

[77] Yu L Lin YL Yan M Li T Wu EY Zimmel K. Hyaline cartilage differentiation of fibroblasts in regeneration and regenerative medicine. Development. (2022) 149(2):dev200249. doi: 10.1242/dev.200249 35005773 PMC8917415

[78] Penn JW Grobbelaar AO Rolfe KJ. The role of the TGF-β family in wound healing, burns and scarring: a review. Int J burns trauma. (2012) 2:18–28. 22928164 PMC3415964

[79] Aloise AC Pereira MD Duailibi SE Gragnani A Ferreira LM. TGF-β1 on induced osteogenic differentiation of human dermal fibroblast. Acta cirurgica brasileira. (2014) 29 Suppl 1:1–6. doi: 10.1590/S0102-86502014001300001 25185048

[80] Yamamoto K Kishida T Nakai K Sato Y Kotani SI Nishizawa Y. Direct phenotypic conversion of human fibroblasts into functional osteoblasts triggered by a blockade of the transforming growth factor-β signal. Sci Rep. (2018) 8:8463. doi: 10.1038/s41598-018-26745-2 29855543 PMC5981640

[81] Maki RG. Small is beautiful: insulin-like growth factors and their role in growth, development, and cancer. J Clin Oncol. (2010) 28:4985–95. doi: 10.1200/JCO.2009.27.5040 PMC3039924 20975071

[82] Tahimic CG Wang Y Bikle DD. Anabolic effects of IGF-1 signaling on the skeleton. Front endocrinology. (2013) 4:6. doi: 10.3389/fendo.2013.00006 PMC3563099 23382729

[83] Ge P Cui Y Liu F Luan J Zhou X Han J. L-carnitine affects osteoblast differentiation in NIH3T3 fibroblasts by the IGF-1/PI3K/Akt signalling pathway. Biosci Trends. (2015) 9:42–8. doi: 10.5582/bst.2015.01000 25787908

[84] Lu Z Chiu J Lee LR Schindeler A Jackson M Ramaswamy Y. Reprogramming of human fibroblasts into osteoblasts by insulin-like growth factor-binding protein 7. Stem Cells Transl Med. (2020) 9:403–15. doi: 10.1002/sctm.19-0281 PMC7031646 31904196

[85] Huang X Qu R Peng Y Yang Y Fan T Sun B. Mechanical sensing element PDLIM5 promotes osteogenesis of human fibroblasts by affecting the activity of microfilaments. Biomolecules. (2021) 11(5):759. doi: 10.3390/biom11050759 34069539 PMC8161207

[86] Li DH He CR Liu FP Li J Gao JW Li Y. Annexin A2, up-regulated by IL-6, promotes the ossification of ligament fibroblasts from ankylosing spondylitis patients. BioMed Pharmacother. (2016) 84:674–9. doi: 10.1016/j.biopha.2016.09.091 27697640

[87] Li F He M Li S Bai Y. Combination of parathyroid hormone pretreatment and mechanical stretch promotes osteogenesis of periodontal ligament fibroblasts. Am J Orthod Dentofacial Orthop. (2022) 161:e62–71. doi: 10.1016/j.ajodo.2021.04.023 34663539

[88] Montgomery SR Nargizyan T Meliton V Nachtergaele S Rohatgi R Stappenbeck F. A novel osteogenic oxysterol compound for therapeutic development to promote bone growth: activation of hedgehog signaling and osteogenesis through smoothened binding. J Bone Miner Res. (2014) 29:1872–85. doi: 10.1002/jbmr.2213 PMC4457783 24591126

[89] Ogisu K Fujio M Tsuchiya S. Conditioned media from mesenchymal stromal cells and periodontal ligament fibroblasts under cyclic stretch stimulation promote bone healing in mouse calvarial defects. Cytotherapy. (2020) 22:543–51. doi: 10.1016/j.jcyt.2020.05.008 32798177

[90] Li Z Zheng J Wan D Yang X. Uniaxial static strain promotes osteoblast proliferation and bone matrix formation in distraction osteogenesis *in vitro* . BioMed Res Int. (2020) 2020:3906426. doi: 10.1155/2020/3906426 32855965 PMC7443025

[91] Weiss S Baumgart R Jochum M Strasburger CJ Bidlingmaier M. Systemic regulation of distraction osteogenesis: a cascade of biochemical factors. J Bone Miner Res. (2002) 17:1280–9. doi: 10.1359/jbmr.2002.17.7.1280 12096842

[92] Yeung HY Lee KM Fung KP Leung KS. Sustained expression of transforming growth factor-beta1 by distraction during distraction osteogenesis. Life Sci. (2002) 71:67–79. doi: 10.1016/S0024-3205(02)01575-8 12020749

[93] Stains JP Watkins MP Grimston SK Hebert C Civitelli R. Molecular mechanisms of osteoblast/osteocyte regulation by connexin43. Calcified Tissue Int. (2014) 94:55–67. doi: 10.1007/s00223-013-9742-6 PMC3815501 23754488

[94] Yang HS Lu XH Chen DY Yuan W Yang LL Chen Y. Mechanical strain induces Cx43 expression in spinal ligament fibroblasts derived from patients presenting ossification of the posterior longitudinal ligament. Eur Spine J. (2011) 20:1459–65. doi: 10.1007/s00586-011-1767-9 PMC3175908 21442291

[95] Li S Zhang H Li S Yang Y Huo B Zhang D. Connexin 43 and ERK regulate tension-induced signal transduction in human periodontal ligament fibroblasts. J Orthop Res. (2015) 33:1008–14. doi: 10.1002/jor.22830 25731708

[96] Julien A Kanagalingam A Martínez-Sarrà E Megret J Luka M Ménager M. Direct contribution of skeletal muscle mesenchymal progenitors to bone repair. Nat Commun. (2021) 12:2860. doi: 10.1038/s41467-021-22842-5 34001878 PMC8128920

[97] Phillips AM. Overview of the fracture healing cascade. Injury. (2005) 36 Suppl 3:S5–7. doi: 10.1016/j.injury.2005.07.027 16188551

[98] Schindeler A McDonald MM Bokko P Little DG. Bone remodeling during fracture repair: The cellular picture. Semin Cell Dev Biol. (2008) 19:459–66. doi: 10.1016/j.semcdb.2008.07.004 18692584

[99] Long F. Building strong bones: molecular regulation of the osteoblast lineage. Nat Rev Mol Cell Biol. (2011) 13:27–38. doi: 10.1038/nrm3254 22189423

[100] Alford AI Hankenson KD. Matricellular proteins: Extracellular modulators of bone development, remodeling, and regeneration. Bone. (2006) 38:749–57. doi: 10.1016/j.bone.2005.11.017 16412713

[101] Baroli B. From natural bone grafts to tissue engineering therapeutics: Brainstorming on pharmaceutical formulative requirements and challenges. J Pharm Sci. (2009) 98:1317–75. doi: 10.1002/jps.21528 18729202

[102] Galea GL Zein MR Allen S Francis-West P. Making and shaping endochondral and intramembranous bones. Dev dynamics. (2021) 250:414–49. doi: 10.1002/dvdy.278 PMC7986209 33314394

[103] Matthews BG Novak S Sbrana FV Funnell JL Cao Y Buckels EJ. Heterogeneity of murine periosteum progenitors involved in fracture healing. eLife. (2021) 10:e58534. doi: 10.7554/eLife.58534 33560227 PMC7906599

[104] Sakai D Mochida J Iwashina T Hiyama A Omi H Imai M. Regenerative effects of transplanting mesenchymal stem cells embedded in atelocollagen to the degenerated intervertebral disc. Biomaterials. (2006) 27:335–45. doi: 10.1016/j.biomaterials.2005.06.038 16112726

[105] Nam YJ Song K Luo X Daniel E Lambeth K West K. Reprogramming of human fibroblasts toward a cardiac fate. Proc Natl Acad Sci United States America. (2013) 110:5588–93. doi: 10.1073/pnas.1301019110 PMC3619357 23487791

[106] Huang Y Tan S. Direct lineage conversion of astrocytes to induced neural stem cells or neurons. Neurosci bulletin. (2015) 31:357–67. doi: 10.1007/s12264-014-1517-1 PMC5563687 25854678

[107] Lai S Zhang M Xu D Zhang Y Qiu L Tian C. Direct reprogramming of induced neural progenitors: a new promising strategy for AD treatment. Trans neurodegeneration. (2015) 4:7. doi: 10.1186/s40035-015-0028-y PMC4422611 25949812

[108] Mizoshiri N Kishida T Yamamoto K Shirai T Terauchi R Tsuchida S. Transduction of Oct6 or Oct9 gene concomitant with Myc family gene induced osteoblast-like phenotypic conversion in normal human fibroblasts. Biochem Biophys Res Commun. (2015) 467:1110–6. doi: 10.1016/j.bbrc.2015.10.098 26499074

[109] Yamamoto K Sato Y Honjo K Ichioka H Oseko F Sowa Y. Generation of directly converted human osteoblasts that are free of exogenous gene and xenogenic protein. J Cell Biochem. (2016) 117:2538–45. doi: 10.1002/jcb.25546 26990860

[110] Wang H Yang Y Liu J Qian L. Direct cell reprogramming: approaches, mechanisms and progress. Nat Rev Mol Cell Biol. (2021) 22:410–24. doi: 10.1038/s41580-021-00335-z PMC8161510 33619373

[111] Zhu H Swami S Yang P Shapiro F Wu JY. Direct reprogramming of mouse fibroblasts into functional osteoblasts. J Bone Miner Res. (2020) 35:698–713. doi: 10.1002/jbmr.3929 31793059 PMC11376108

[112] Phillips JE Garcia AJ. Retroviral-mediated gene therapy for the differentiation of primary cells into a mineralizing osteoblastic phenotype. Methods Mol Biol. (2008) 433:333–54. doi: 10.1007/978-1-59745-237-3_20 18679633

[113] Ahmed MF El-Sayed AK Chen H Zhao R Jin K Zuo Q. Direct conversion of mouse embryonic fibroblast to osteoblast cells using hLMP-3 with Yamanaka factors. Int J Biochem Cell Biol. (2019) 106:84–95. doi: 10.1016/j.biocel.2018.11.008 30453092

[114] Sommar P Pettersson S Ness C Johnson H Kratz G Junker JP. Engineering three-dimensional cartilage- and bone-like tissues using human dermal fibroblasts and macroporous gelatine microcarriers. J plastic reconstructive aesthetic Surg JPRAS. (2010) 63:1036–46. doi: 10.1016/j.bjps.2009.02.072 19329368

[115] Dolanmaz D Saglam M Inan O Dundar N Alniacık G Gursoy Trak B. Monitoring bone morphogenetic protein-2 and -7, soluble receptor activator of nuclear factor-κB ligand and osteoprotegerin levels in the peri-implant sulcular fluid during the osseointegration of hydrophilic-modified sandblasted acid-etched and sandblasted acid-etched surface dental implants. J periodontal Res. (2015) 50:62–73. doi: 10.1111/jre.12182 24697526

